# Alzheimer's disease pathology: pathways between chronic vascular risk factors and blood-brain barrier dysfunction in a cohort of patients with different types of dementia

**DOI:** 10.3389/fnagi.2023.1088140

**Published:** 2023-05-04

**Authors:** Jinghuan Gan, Xia Yang, Guili Zhang, Xudong Li, Shuai Liu, Wei Zhang, Yong Ji

**Affiliations:** ^1^Department of Neurology, Beijing Tiantan Hospital, Capital Medical University, Beijing, China; ^2^Department of Cognitive Disorder, Beijing Tiantan Hospital, Capital Medical University, Beijing, China; ^3^China National Clinical Research Center for Neurological Diseases, Beijing, China; ^4^Department of Neurology, Tianjin Dementia Institute, Tianjin Key Laboratory of Cerebrovascular and Neurodegenerative Diseases, Tianjin Huanhu Hospital, Tianjin, China

**Keywords:** blood-brain barrier, Qalb, Alzheimer's disease, dementia, diabetes mellitus

## Abstract

**Background:**

Blood brain barrier (BBB) breakdown is considered a potential mechanism of dementia. The Alzheimer's disease (AD) biomarkers and vascular factors are also associated with BBB permeability.

**Objective:**

In the present study, the combination effects of neuropathological biomarkers of AD and chronic vascular risk factors for BBB were investigated.

**Methods:**

The cerebrospinal fluid (CSF)/serum albumin ratio (Qalb), an indicator of BBB permeability, was measured in a total of 95 hospitalized dementia patients. The demographics, clinical information, and laboratory tests were collected from the inpatient records. The CSF neuropathological biomarkers of AD and apolipoprotein E (APOE) genotype were also collected. The mediation analysis model was used to calculate the associations among neuropathological biomarkers of AD (mediator), the Qalb, and chronic vascular risk factors.

**Results:**

Three types of dementia, AD (*n* = 52), Lewy body dementia (LBD, *n* = 19), and frontotemporal lobar degeneration (*n* = 24), were included with a mean Qalb of 7.18 (± 4.36). The Qalb was significantly higher in dementia patients with type 2 diabetes mellitus (T2DM, *p* = 0.004) but did not differ based on the presence of APOE ε4 allele, CMBs, or amyloid/tau/neurodegeneration (ATN) framework. The Qalb was negatively associated with the levels of Aβ1-42 (B = −20.775, *p* = 0.009) and Aβ1-40 (B = −305.417, *p* = 0.005) and positively associated with the presence of T2DM (B = 3.382, *p* < 0.001) and the levels of glycosylated hemoglobin (GHb, B = 1.163, *p* < 0.001) and fasting blood glucose (FBG, B = 1.443, *p* < 0.001). GHb is a direct chronic vascular risk factor for higher Qalb (total effect B = 1.135, 95% CI: 0.611–1.659, *p* < 0.001). Ratios of Aβ1-42/Aβ1-40 or t-tau/Aβ1-42 were mediators of the association between the Qalb and GHb; the direct effect of GHb on the Qalb was 1.178 (95% CI: 0.662–1.694, *p* < 0.001).

**Conclusion:**

Glucose exposure can directly or indirectly affect BBB integrity through Aβ and tau, indicating glucose affects BBB breakdown and glucose stability plays an important role in dementia protection and management.

## Introduction

The blood-brain barrier (BBB) is a selective diffusion barrier to separate the central nervous system (CNS) from the peripheral blood circulation and maintains homeostasis in the CNS by regulating ion balance, facilitating nutritional transport, and preventing influx of potentially neurotoxic molecules from circulation (Kadry et al., 2020). BBB damage has been commonly observed and can be reflected in alterations in neuroimaging (Chagnot et al., 2021) and biofluid markers (Wong et al., 2022), indicating the important role of BBB in dementia (Raja et al., 2018).

Literature based on post-mortem investigations and dynamic contrast-enhanced magnetic resonance imaging (DCE-MRI) studies have shown significant BBB damage in normal elderly adults (Verheggen et al., 2020) and dementia patients (Sweeney et al., 2018). The increased regional BBB K*trans* (a non-invasive indicator of BBB permeability) in the global cortex (van de Haar et al., 2016), median temporal lobe or hippocampus (Montagne et al., 2019), and white matter (Kerkhofs et al., 2021) is common in patients with cognitive impairment based on DCE-MRI (Raja et al., 2018). Biofluid markers (Chen, 2011; Sun et al., 2021; Wong et al., 2022), including serum levels of the S100 calcium binding protein B, matrix metalloproteinases, glial fibrillary acidic protein, neurofilament light chain protein, soluble platelet-derived growth factor β (sPDGFRβ), and the cerebrospinal fluid (CSF)/serum albumin ratio (Qalb) (Wong et al., 2022), have appropriate but varying levels of sensitivity and specificity. The alteration of Qalb is regarded a reliable standard surrogate marker for BBB integrity and a potential biomarker for neurological diseases. The Qalb was found increased in patients with Parkinson's disease (Pisani et al., 2012), Alzheimer's disease (AD), and vascular dementia (VaD) (Musaeus et al., 2020) compared with healthy individuals, as well as in a small subset of patients with progressive supranuclear palsy, multiple system atrophy, and Lewy body dementia (LBD) (Llorens et al., 2015). Aging (Montagne et al., 2019; Verheggen et al., 2020), gender (Moon Y. et al., 2021), and apolipoprotein E (APOE) ε4 allele (Montagne et al., 2020) are typical factors associated with BBB integrity. The neuropathological biomarkers of AD (Wong et al., 2022) and chronic vascular risk factors such as cerebral microbleeds (CMBs), enlarged perivascular spaces, type 2 diabetes mellitus (T2DM), arterial hypertension, dyslipidemia, and hyperhomocysteinemia (HHcy), also contribute to increased BBB permeability in dementia (Wang et al., 2018; Li et al., 2019; Freeze et al., 2020; Cai et al., 2021). The Qalb is not consistently altered in any neurodegenerative dementia (Musaeus et al., 2020) and the association among the Qalb, APOE ε4 allele, and neuropathological biomarkers of AD are controversial (Karch et al., 2013; Janelidze et al., 2017). In a preliminary analysis, BBB permeability was associated with dementia and vascular risk factors but not amyloid pathology or APOE genotype (Janelidze et al., 2017). Clear evidence exists for the independent role of vascular risk factors or AD neuropathological biomarkers in the pathogenesis of BBB dysfunction, however, chronic vascular risk factors mediated by AD neuropathological biomarkers that contribute to BBB dysfunction in AD and other forms of dementia cannot be excluded.

To evaluate the Qalb in Chinese dementia patients and explore the combined effects of neuropathological biomarkers of AD and chronic vascular risk factors for BBB, the Qalb, β-amyloid (Aβ), and tau levels in CSF were analyzed for neurodegenerative dementias. These findings will contribute to the understanding of disease mechanisms of dementia and facilitate development of precise intervention and management measures for chronic vascular factors to prevent dementia.

## Materials and methods

This study was performed according to the Helsinki Declaration and approved by the Ethical Review Board of Beijing Tiantan Hospital (KYSQ 2021-068-01). Written informed consents was obtained from patients and their family members. All methods were performed following relevant guidelines and regulations.

### Patients

The study included 95 hospitalized patients recruited from the department of cognitive disorders of Beijing Tiantan Hospital, Capital Medical University from December 2021 to June 2022, diagnosed with AD (n = 52), LBD (*n* = 19), or frontotemporal lobar degeneration (FTLD, *n* = 24). Probable AD was diagnosed according to the criteria of the National Institute on Aging and the Alzheimer's Association (NIA-AA) workgroup and ^11^C-PIB PET scans to assess Aβ deposition (*n* = 7) or CSF test for neuropathological biomarkers of AD (*n* = 52) (McKhann et al., 2011). Consensus criteria for the diagnosis of FTLD were formulated in 1998 (Neary et al., 1998). LBD subjects included patients with dementia with Lewy bodies (DLBs) and Parkinson's disease dementia (PDD); patients with probable DLB were diagnosed using the criteria of McKeith et al. (2017), and probable PDD was diagnosed according to the clinical criteria developed by the Movement Disorder Society in Emre et al. (2007). A probable DLB diagnosis can be made with two or more core symptoms together with or without indicative biomarkers, or only one core symptom with one or more indicative biomarkers. International consensus suggests DLB should be diagnosed when cognitive impairment precedes parkinsonism or begins within a year of parkinsonism and PDD should be diagnosed when parkinsonism precedes cognitive impairment by more than 1 year.

### Exclusion criteria for enrolled patients

- The patients who had diagnosis of any neurological disease except AD, LBD, or FTLD;
- The patients could not cooperate with lumbar puncture, MRI, and cognitive evaluation due to various reasons were excluded.
- The patients had the history of mental disorders and illicit drug abuse;
- The patients were treated with folate or vitamin B12 in the last 3 months;
- The patients had acute or chronic liver and kidney dysfunction, malignant tumors, or other serious underlying diseases.

### Clinical information

The general demographics of each patient, including gender, age, body mass index (BMI), educational level, and blood laboratory tests performed on the day of hospital admission (fasting blood glucose (FBG), glycosylated hemoglobin (GHb), triglyceride, cholesterol, high-density lipoprotein cholesterol (HDL-C), low-density lipoprotein cholesterol (LDL-C), homocysteine (Hcy), serum folate, serum vitamin B12, and ferritin), were obtained from inpatient medical records.

The clinical and neurological evaluations were performed by neurologists specialized in dementia care. A detailed history taken from the primary caregivers of the patient included the history of hypertension, T2DM, hyperlipidemia (HLP), HHcy, cardio-cerebrovascular disease (CVD), smoking habits and/or alcohol consumption, course of disease, and prescriptions for patients in the last 3 months. Before lumbar puncture, the blood pressure was measured twice on the right upper arm using an electronic blood pressure monitor (Omron HEM-7430; Omron Corporation, Kyoto, Japan) with 1 min between measurements. The mean values of systolic blood pressure and diastolic blood pressure were calculated and recorded. If the difference between the two blood pressure readings exceeded 10 mmHg, a third measurement was taken and the mean value of the last two readings was calculated (Lu et al., 2017).

### Neuropsychological assessments

Neuropsychological assessments were performed on the same day as the lumbar puncture. The Mini-Mental State Examination-Chinese version (C-MMSE) (Folstein et al., 1975), the Montreal Cognitive Assessment (MoCA) (Nasreddine et al., 2005), and the Clinical Dementia Rating (CDR) (Morris, 1993) scale were used to evaluate global cognitive function and severity of cognitive impairment in all patients. The C-MMSE and MoCA scores range from 0 (severe impairment) to 30 (no impairment). CDR is a 5-point scale; 0.0 (no dementia), 0.5 (MCI), 1.0 (mild), 2.0 (moderate), and 3.0 (severe).

### Sample collection and measurements

Blood samples were drawn by venipuncture into 6-mL plastic vacuum tubes containing EDTA on the day of hospital admission; CSF was collected *via* a lumbar puncture in the L3–L5 vertebral interspaces between 7 a.m. and 10 a.m. after fasting. Then, all samples were centrifuged, aliquoted, and stored at −80°C in polypropylene tubes until use. Anti-AD drugs were withheld for 12–14 h prior to sampling the CSF, and the gap between blood and CSF collection was within 48 h.

All analyses of blood and CSF samples were performed using commercial and validated instruments and kits at the Clinical Neurochemistry Laboratory at Beijing Tiantan Hospital, Beijing, China. Serum albumin levels were analyzed using the absorption method and CSF albumin levels analyzed using an immunoturbidimetric assay. The Qalb was used to reflect BBB permeability. CSF Aβ1-42 (RE59661, IBL International, Hamburg, Germany), Aβ1-40 (RE59651, IBL International, Hamburg, Germany), t-tau (RE59631, IBL International, Hamburg, Germany), and p-tau181 (30121609, IBL International, Hamburg, Germany) concentrations were quantified using commercial enzyme-linked immunosorbent assays (ELISAs) according to the manufacturer's protocol. CSF cut-off values for Aβ-positive or Aβ-negative were Aβ1-42 < 550 pg/mL and/or Aβ1-42/Aβ1-40 ratio ≤ 0.05. CSF cut-off values for tau positive were p-tau181 > 50 pg/mL and/or t-tau > 399 pg/mL, all cut-off values were set based on the accumulation of previous experimental data of Kindstar Global Genetic Technology Co., LTD. APOE genotype was determined based on genomic DNA using polymerase chain reaction following the detailed protocol described in our previous study (Gan et al., 2022).

#### MRI acquisition and visual rating scales

Multiplanar oblique coronal (perpendicular to the axis of the hippocampus), transverse, and coronal position reconstructions were made of 3D T1-weighted images for diagnostic multisequence MRI; details of the protocol are provided in our previous study (Zhu et al., 2021; Gan et al., 2022). All MRI visual scales readings were reviewed by two experienced neuroradiologists in a double-blind manner and the final rating scores averaged.

The visual rating scales included Medial Temporal Lobe Atrophy (MTA) and Fazekas scales. MTA is used to evaluate the visual regional brain atrophy in the hippocampus, parahippocampal gyrus, entorhinal cortex, and the surrounding CSF spaces, with a range from 0 (no atrophy) to 4 (severe loss of hippocampal volume) (Scheltens et al., 1992). Fazekas scales reflect the whole white matter lesion and range from 0 (no or single punctate lesion) to 3 (large confluent lesions) (Fazekas et al., 1987). The CMBs were defined as round or quasi-round areas with clear boundaries and black or low signal areas with a diameter of 2–10 mm in the T2 gradient echo sequence or SWI (Greenberg et al., 2009).

**Figure 1:**
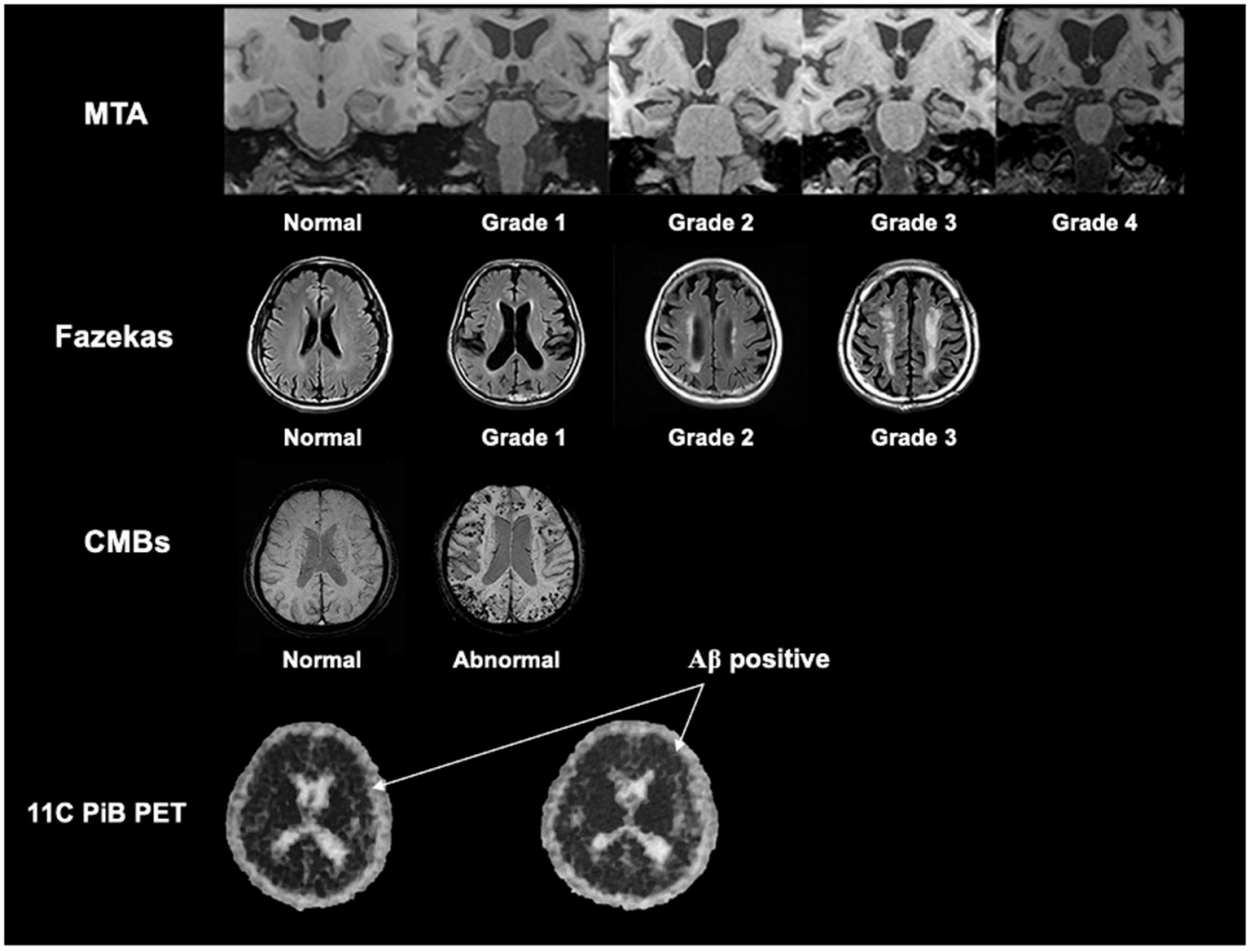
The reconstruction mode and the description of the degree of the MRI visual rating scales. We referred our previous study by Zhu et al. (2021), and showed the reconstruction mode and the description of the degree of the MTA scale, Fazekas scale, and CMBs based on MRI. We also showed the rep images of Aβ positive in 11C-PiB PET (the black dot in the skull indicated by the arrow is positive). MTA, medial temporal lobe atrophy; CMBs, cerebral microbleeds; Aβ, β-amyloid.

The reconstruction mode and the degree of the MRI visual rating scales were used as described in our previous study (Zhu et al., 2021) and shown in Figure 1.

### Statistical analysis

The clinical information, including general demographics, medical history, course of disease, blood pressure, neuropsychological assessments, blood laboratory tests, and neuropathological biomarker levels of AD in CSF, were collected. For quantitative variables, if the data satisfied the normal distribution, the mean (standard deviation, SD) was described, and comparison among different dementia groups was performed using the *t*-test; if the data did not satisfy the normal distribution, the data were described as the medians (interquartile range, IQR), and comparison among different groups performed using the Mann–Whitney *U* test. The qualitative variables were expressed as frequency and the chi-squared test used for comparison among different groups. The demographic and clinical information are expressed as mean (SD) and shown in Table 1. The participants with APOE ε2/ε2, APOE ε2/ε3, or APOE ε3/ε3 were classified into the APOE ε4 non-carrier group and subjects with APOE ε2/ε4, APOE ε3/ε4, or APOE ε4/ε4 were classified into the APOE ε4 carrier group.

**Table 1:** Demographic features and laboratory tests in dementia cases.

	**All patients (*n* = 95)**	**AD (*n* = 52)**	**LBD (*n* = 19)**	**FTLD (*n* = 24)**
**Gender**				
Male	41 (43.2%)	21 (40.4%)	10 (52.6%)	10 (41.7%)
Female	54 (56.8%)	31 (59.6%)	9 (47.4%)	14 (58.3%)
Age (years old)	65.53 ± 9.49	63.75 ± 9.85	68.05 ± 9.48	67.38 ± 8.18
Education (years)	8.60 ± 4.52	8.29 ± 4.76	9.16 ± 3.89	8.83 ± 4.55
BMI (kg/m^2^)	23.25 ± 3.61	22.96 ± 3.27	22.93 ± 4.08	24.15 ± 3.92
Course of disease (months)	35.74 ± 36.03	41.23 ± 43.58	35.26 ± 26.70	24.21 ± 18.14
**Chronic vascular risk factors (** ***n*** **, %)**				
Habits of SA	26 (27.4%)	15 (28.8%)	5 (26.3%)	6 (25.5%)
Hypertension	27 (28.4%)	12 (23.1%)	6 (31.6%)	9 (37.5%)
CVD	15 (15.8%)	5 (9.6%)	4 (21.1%)	6 (25.0%)
Type 2 diabetes mellitus	24 (25.3%)	13 (25.0%)	6 (31.6%)	5 (20.8%)
Hyperlipidemia	33 (34.7%)	22 (42.3%)	6 (31.6%)	5 (20.8%)
Hyperhomocysteinemia	36 (37.9%)	21 (40.4%)	8 (42.1%)	7 (29.2%)
APOE ε4 carriers, *n* (%)	19 (20.0%)	11 (21.2%)	5 (26.3%)	3 (12.5%)
**Neuropsychological assessments**				
MMSE	11.78 ± 6.36	11.15 ± 6.56	12.68 ± 5.83	12.42 ± 6.43
MoCA	7.62 ± 4.61	7.35 ± 4.93	8.05 ± 3.92	7.88 ± 4.52
CDR	2.02 ± 0.76	2.08 ± 0.74	1.89 ± 0.81	2.00 ± 0.78
**MRI assessment**				
MTA (Left)	1.82 ± 0.92	1.81 ± 0.95	1.95 ± 0.91	1.75 ± 0.90
MTA (Right)	1.80 ± 0.92	1.75 ± 0.93	2.05 ± 0.97	1.71 ± 0.86
Fazekas scores	1.66 ± 0.77	1.63 ± 0.74	1.47 ± 0.70	1.88 ± 0.85
CMBs, n (%)	21 (22.1%)	12 (23.1%)	3 (15.8%)	6 (25.0%)
**Laboratory tests**				
FBG (mmol/L)	5.09 ± 1.30	5.06 ± 1.18	4.98 ± 1.06	5.26 ± 4.57
Glycosylated hemoglobin (%)	6.28 ± 1.49	6.30 ± 1.60	6.35 ± 1.33	6.18 ± 1.41
Homocysteine (μmol/L)	15.48 ± 7.18	15.90 ± 7.93	15.86 ± 6.61	14.25 ± 5.94
Folate (ng/mL)	7.62 ± 4.89	7.86 ± 5.08	6.51 ± 4.83	7.99 ± 4.56
Vitamin B_12_ (pg/mL)	536.67 ± 427.91	488.75 ± 324.45	514.68 ± 436.20	657.92 ± 587.44
Ferritin (ng/mL)	140.46 ± 146.80	124.72 ± 99.27	177.23 ± 131.41	145.48 ± 225.86
Triglyceride (mmol/L)	1.27 ± 0.73	1.34 ± 0.88	1.23 ± 0.50	1.14 ± 0.50
Cholesterol (mmol/L)	4.71 ± 1.16	4.96 ± 1.05	4.63 ± 1.39	4.21 ± 1.06^*^
HDL-c (mmol/L)	1.39 ± 0.41	1.45 ± 0.49	1.30 ± 0.26	1.33 ± 0.31
LDL-c (mmol/L)	6.03 ± 30.81	8.83 ± 41.60	2.86 ± 1.34	2.47 ± 0.99^*^
S-ALB	39.63 ± 4.47	40.22 ± 4.31	39.08 ± 4.92	38.79 ± 4.46
S-IgG	11.81 ± 2.09	11.69 ± 1.69	12.26 ± 2.78	11.71 ± 2.30
CSF-24h/IgG	2.44 ± 6.02	2.87 ± 7.49	1.51 ± 3.18	2.26 ± 3.84
CSF-ALB	0.28 ± 0.17	0.29 ± 0.20	0.24 ± 0.08	0.30 ± 0.14
CSF-IgG	0.04 ± 0.03	0.04 ± 0.04	0.04 ± 0.01	0.04 ± 0.02
Qalb	7.18 ± 4.36	7.24 ± 5.11	6.31 ± 2.44	7.71 ± 3.77

Unless otherwise indicated, values are presented as mean ± standard deviation. *P* < 0.017 (Bonferroni correction) was considered statistically significant. ^*^ There were significant differences between AD and FTLD groups in cholesterol (*p* = 0.013) and LDL (*p* = 0.014) levels.

AD, Alzheimer's disease; LBD, Lewy body dementia; FTLD, frontotemporal lobar degeneration; BMI, body mass index; SA, habit of smoking and/or alcohol consumption; CVD, cardiac-cerebral vascular disease; APOE, Apolipoprotein E; MMSE, Mini-Mental State Examination; MoCA, the Montreal Cognitive Assessment; CDR, the clinical dementia rating; MRI, Magnetic Resonance Imaging; MTA, medial temporal lobe atrophy; CMBs, cerebral microbleeds; FBG, fasting blood glucose; HDL-c, high-density lipoprotein cholesterol; LDL-c, low-density lipoprotein cholesterol; S-ALB, serum albumin; S-IgG, serum immunoglobulin G; CSF-24h/IgG, 24h cerebrospinal fluid immunoglobulin G; CSF-ALB, cerebrospinal fluid albumin; CSF-IgG, cerebrospinal fluid immunoglobulin G; Qalb, CSF/serum albumin quotient.

The partial correlation and linear regressions, after adjusting for gender, age, educational level, course of disease and diagnosis, were used to analyze the association among the Qalb, neuropathological biomarkers of AD, and chronic vascular risk factors in dementia patients. Then, to test our hypotheses that neuropathological biomarkers of AD in CSF could act as a mediator of the association between the Qalb and chronic vascular risk factors, generalized structural equation models were constructed. Because significant differences were not observed in blood pressure, CVD, blood lipids, blood Hcy, and smoking habits and/or alcohol consumption, the association among the exposure (T2DM, GHb, and FBG), mediators (the ratios of Aβ1-42/Aβ1-40 or t-tau/Aβ1-42), and outcome (Qalb) was analyzed using linear regression after adjusting for gender, age, course of disease, APOE ε4 status, and diagnosis. For all pathways, standardized direct, specific indirect, and total indirect were estimated.

Statistical analysis was performed using the IBM SPSS (version 26.0; IBM Corporation, Armonk, NY, USA). *P*-values < 0.05 were considered statistically significant at the 2-tailed α level, and comparison among the three groups (AD, LBD, and FTLD) was controlled using Bonferroni correction.

## Results

### Sample characteristics

Demographic and clinical characteristic are shown in Table 1. Among the 95 patients enrolled, 54 (56.8%) were female and the average age was 65.53 years (± 9.49 years). The mean Qalb was 7.18 (± 4.36). Except for the cholesterol and LDL-C levels, significant difference was not found in gender, age, course of disease, educational level, BMI, CVD, neuropsychological assessments, MTA and Fazekas scores, presence of CMBs, and other laboratory tests among the three groups.

### Comparison of Qalb and neuropathological biomarkers of AD

The Qalb and Aβ1-42, Aβ1-40, p-tau181, and t-tau levels in CSF were measured and compared based on different chronic vascular risk factors (Table 2). In patients with a history of T2DM, the median (IQR) Qalb [7.36 (5.63–14.21) *vs*. 5.61 (4.50–6.99), *p* = 0.004] was significantly higher, and CSF Aβ1-40 [7,429.46 pg/mL (5,558.52–10,299.55 pg/mL) *vs*. 10,952.22 pg/mL (8,429.93–1,3315.14 pg/mL), *p* = 0.010] and t-tau [386.33 pg/mL (257.25–606.50 pg/mL *vs*. 537.75 pg/mL (412.00–700.45 pg/mL), *p* = 0.015] were significantly lower than in subjects without a history of T2DM. In addition, the Aβ1-40 levels were significantly decreased in patients with smoking habits and/or alcohol consumption (*p* = 0.028) compared with subjects who did not smoke and/or consume alcohol. Significant differences were not found in the Qalb and Aβ1-42, Aβ1-40, p-tau181, and t-tau levels in hypertension, CVD, HLP, HHcy, and APOE ε4 allele groups.

**Table 2:** The median level of Qalb and CSF AD neuropathological biomarkers according to chronic vascular risk factors.

**Chronic vascular risk factors**		**Qalb**	**Aβ1-42 (pg/ml)**	**Aβ1-40 (pg/ml)**	**p-tau181p (pg/ml)**	**t-tau (pg/ml)**	**Aβ1-42/Aβ1-40**	**t-tau/Aβ1-42**
Hypertension	Without (*n* = 68)	5.72 (4.54, 7.23)	469.60 (311.96, 614.15)	10,233.89 (6,076.93, 13,044.38)	57.95 (43.25, 80.75)	506.76 (374.39, 645.56)	0.05 (0.04, 0.07)	1.10 (0.68 2.01)
	With (*n* = 27)	6.76 (4.79, 9.03)	545.64 (345.79, 902.08)	9,680.74 (8,447.45, 13,174.44)	56.61 (42.26, 67.03)	540.67 (389.21, 718.60)	0.05 (0.04, 0.08)	0.98 (0.53, 1.55)
	Z-score	−1.135	−1.692	−0.734	−0.021	−0.355	−1.143	−1.493
	*P*-value	0.257	0.091	0.463	0.984	0.723	0.253	0.135
T2DM	Without (*n* = 74)	5.61 (4.50, 6.99)	492.85 (345.79, 678.95)	10,952.22 (8,429.93, 13,315.14)	59.24 (43.76, 80.01)	537.75 (412.00, 700.45)	0.05 (0.04, 0.07)	1.08 (0.68, 1.81)
	With (*n* = 21)	7.36 (5.63, 14.21)	361.99 (218.28, 647.49)	7,429.46 (5,558.52, 10,299.55)	54.77 (36.21, 60.02)	386.33 (257.25, 606.50)	0.05 (0.04, 0.06)	1.00 (0.51, 1.64)
	Z-score	−2.912	−1.439	−2.569	−1.507	−2.424	−0.163	−0.856
	*P*-value	0.004	0.150	0.010	0.132	0.015	0.871	0.392
CVD	Without (*n* = 80)	5.88 (4.55, 7.96)	462.33 (322.33, 616.08)	9,842.74 (6,525.25, 12,981.47)	57.95 (42.27, 80.75)	509.41 (369.76, 668.19)	0.05 (0.04, 0.07)	1.10 (0.67, 1.79)
	With (*n* = 15)	6.12 (4.62, 7.31)	699.90 (278.35, 1,414.38)	12,537.31 (8,447.45, 13,073.24)	54.70 (50.12, 66.12)	610.33 (431.72, 798.00)	0.05 (0.04, 0.09)	0.83 (0.54, 1.60)
	Z-score	−0.051	−1.939	−1.031	−0.143	−1.368	−1.506	−1.010
	*P*-value	0.959	0.052	0.303	0.886	0.171	0.132	0.312
HLP	Without (*n* = 62)	5.88 (4.52, 8.30)	495.08 (323.46, 826.53)	10,519.91 (6,617.96, 13,234.63)	57.95 (43.39, 69.71)	529.24 (396.31, 749.19)	0.05 (0.04, 0.07)	1.01 (0.57, 1.60)
	With (*n* = 33)	6.20 (4.95, 7.39)	411.84 (264.08, 581.89)	9,635.67 (6,841.04, 11,851.92)	57.24 (42.75, 85.52)	493.99 (370.44, 625.24)	0.05 (0.04, 0.06)	1.13 (0.71, 1.94)
	Z-score	−0.180	−1.587	−0.766	−0.063	−1.157	−1.189	−0.836
	*P*-value	0.857	0.113	0.444	0.950	0.247	0.235	0.403
HHcy	Without (*n* = 59)	6.20 (4.62, 7.86)	480.30 (345.15, 699.90)	9,635.67 (6,421.48, 12,537.31)	57.68 (43.40, 66.29)	517.81 (377.60, 675.34)	0.05 (0.04, 0.07)	1.01 (0.67, 1.53)
	With (n = 36)	5.72 (4.47, 8.31)	455.59 (242.11, 654.14)	10,510.17 (7,516.88, 14,419.32)	58.11 (42.27, 103.59)	506.76 (399.61, 764.20)	0.04 (004, 0.06)	1.28 (0.60, 2.51)
	Z-score	−0.529	−0.813	−0.875	−0.702	−0.173	−1.466	−0.744
	*P*-value	0.597	0.416	0.382	0.483	0.863	0.143	0.457
Habits of SA	Without (*n* = 59)	5.64 (4.55, 7.04)	492.85 (345.17, 703.50)	10,926.83 (7,765.80, 13,261.46)	59.24 (43.58, 89.22)	514.21 (385.20, 709.53)	0.05 (0.04, 0.07)	1.01 (0.69, 1.97)
	With (*n* = 26)	7.21 (4.72, 9.28)	411.85 (235.12, 619.47)	8,824.72 (5,609.41, 11,543.75)	56.82 (40.82, 61.00)	464.91 (351.59, 575.63)	0.05 (0.04, 0.07)	1.15 (0.66, 1.63)
	Z-score	−1.565	−1.319	−2.195	−1.219	−1.452	−0.555	−0.192
	*P*-value	0.118	0.187	0.028	0.223	0.146	0.579	0.848
APOE ε4 allele	Without (*n* = 76)	6.27 (4.58, 8.18)	486.57 (311.96, 694.66)	10,048.02 (5,924.00, 12,898.19)	57.52 (42.27, 66.24)	508.17 (369.76, 672.22)	0.05 (0.04, 0.07)	1.05 (0.57, 1.69)
	With (*n* = 19)	5.21 (4.48, 5.65)	456.00 (345.79, 615.00)	9,913.04 (8,023.33, 13,315.14)	62.01 (49.95, 103.87)	543.69 (412, 700.45)	0.04 (0.03, 0.05)	1.12 (0.77, 1.81)
	Z-score	−1.740	−0.465	−0.744	−1.312	−0.940	−1.801	−0.986
	*P*-value	0.082	0.642	0.457	0.190	0.347	0.072	0.324

CSF, cerebrospinal fluid; Qalb, cerebrospinal fluid/serum albumin quotient; AD, Alzheimer's disease; Aβ, β-amyloid; p-tau181, phosphorylated tau181; t-tau, total tau; T2DM, type 2 Diabetes mellitus; CVD, cardiac-cerebral vascular disease; HLP, hyperlipidemia; HHcy, hyperhomocysteinemia; SA, smoking and/or alcohol consumption; APOE, Apolipoprotein E.

**Figure 2:**
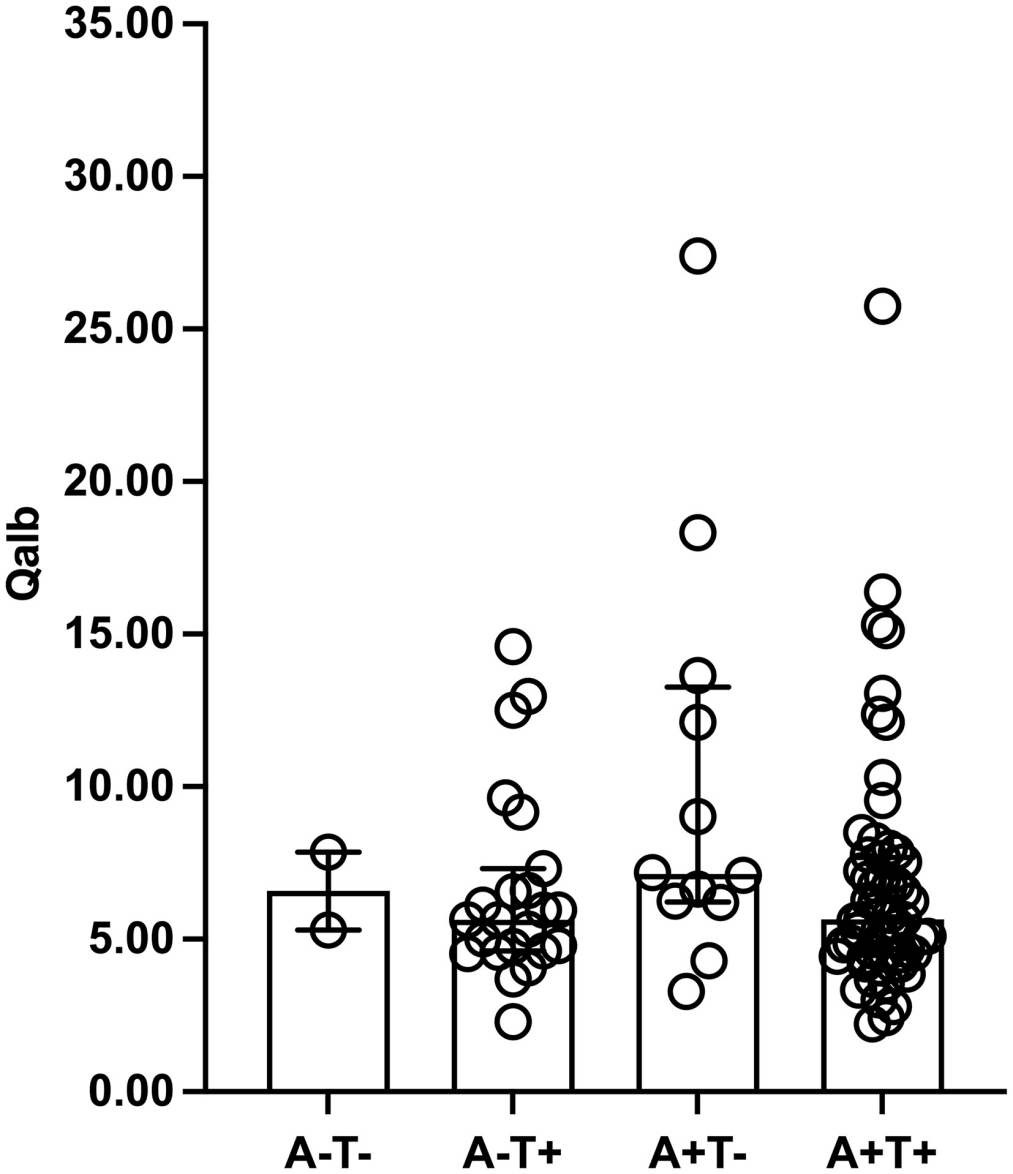
Qalb levels in ATN framework. The median (interquartile range) was used to show the distribution of Qalb in dementia according to ATN framework. The two bars in this finger were Q_25_ and Q_75_ range. Qalb, cerebrospinal fluid/serum albumin quotient; ATN, the Amyloid Tau Neurodegeneration framework.

**Figure 3:**
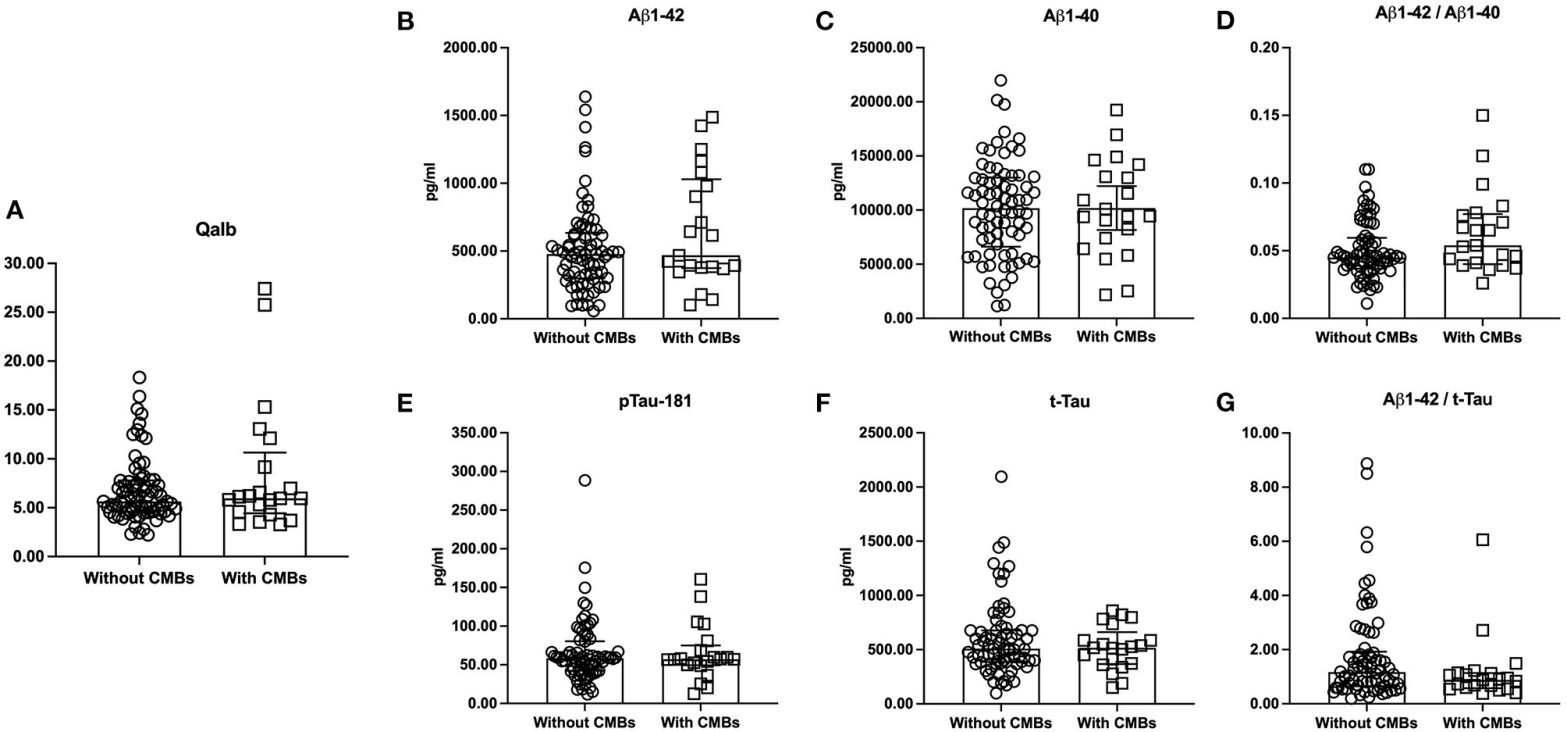
The levels of Qalb and CSF AD neuropathological biomarkers according to CMBs. The medians (interquartile range) were used to show the distribution of Qalb **(A)** and CSF AD neuropathological biomarkers **(B–G)** in dementia according to CMBs. The two bars in these figures were Q_25_ and Q_75_ range. CSF, cerebrospinal fluid; Qalb, cerebrospinal fluid/serum albumin quotient; AD, Alzheimer's disease; CMBs, cerebral microbleeds; Aβ, β-amyloid; p-tau181, phosphorylated tau181; t-tau, total tau.

The amyloid/tau/neurodegeneration (ATN) did not influence Qalb due to the similar Qalb among A–T– [mean ± SD, 6.58 ± 1.80), A–T + [median (IQR) = 5.64 (4.62–7.31)], A+T– (median (IQR) = 7.14 (6.21–13.26)], and A + T + [median (IQR) = 5.65 (4.47–7.68)] patients (*p* = 0.245, Figure 2). In addition, significant differences were not observed in the Qalb and Aβ1-42, Aβ1-40, p-tau181, t-tau levels as well as Aβ1-42/Aβ1-40 and t-tau/Aβ1-42 ratios based on the presence of CMBs (Figure 3).

In A + T + patients with T2DM, the Qalb was increased [with T2DM: 8.24 (5.72–15.21) *vs*. without T2DM: 5.29 (4.29–6.78), *p* = 0.001] but *t*-tau levels were lower [with T2DM: 399.33 pg/mL (230.27–589.49 pg/mL) *vs*. without T2DM: 543.69 pg/mL (438.94–745.80 pg/mL), *p* = 0.027] compared with A + T + patients without T2DM (Table 3). Patients with T2DM had slightly but not significantly higher Qalb than patients without T2DM (A + T– patients with T2DM: 7.18 pg/mL (5.69–18.21 pg/mL) *vs*. without T2DM: 6.64 pg/mL (6.20–13.64 pg/mL), *p* = 0.855; A–T + patients with T2DM: 5.87 pg/mL (4.77–8.57 pg/mL) *vs*. without T2DM: 5.64 pg/mL (4.59–8.24 pg/mL), *p* = 0.726). The levels of Aβ1-42 (*p* = 0.726 in A–T + patients, *p* = 0.100 in A + T– patients), Aβ1-40 (*p* = 0.726 in A–T + patients, *p* = 0.100 in A + T– patients), p-tau181 (*p* = 0.161 in A–T + patients, *p* = 0.068 in A + T– patients), t-tau (*p* = 0.889 in A–T + patients, *p* = 0.361 in A + T– patients), and the ratios of Aβ1-42/Aβ1-40 (*p* = 0.753 in A–T + patients, *p* = 0.314 in A + T– patients) and t-tau/Aβ1-42 (*p* = 0.363 in A–T + patients, *p* = 0.201 in A + T– patients) were similar in A–T + and A + T– patients with and without T2DM.

**Table 3:** Comparisons of Qalb and CSF AD neuropathological biomarkers according to ATN framework and T2DM.

	**A-T-**			**A-T**+		
	**With T2DM (*****n*** = **0)**	**Without T2DM (*****n*** = **2)**	***P*** **-value**	**With T2DM (*****n*** = **6)**	**Without T2DM (*****n*** = **17)**	***P*** **-value**
Qalb	ns	6.58 ±1.80	ns	5.87 (4.77, 8.57)	5.64 (4.59, 8.24)	0.726
Aβ_1 − 42_ (pg/ml)	ns	957.98 ± 394.60	ns	1,078.50 (701.89, 1,439.86)	927.18 (705.18, 1,207.00)	0.726
Aβ_1 − 40_ (pg/ml)	ns	12,329.21 ± 5,582.06	ns	11,675.56 (9,567.95, 14,843.85)	13,072.14 (9,615.91, 14,761.10)	0.726
p-tau181 (pg/ml)	ns	39.61 ± 5.08	ns	55.22 (36.36, 71.04)	60.66 (55.66, 82.76)	0.161
t-tau (pg/ml)	ns	311.25 ± 13.08	ns	609.41 (446.75, 71.04)	585.00 (507.50, 809.61)	0.889
Aβ1-42/Aβ1-40	ns	0.08 ± 0.00	ns	0.07 (0.06, 0.13)	0.08 (0.06, 0.090)	0.753
T-tau/Aβ1-42	ns	0.36 ± 0.16	ns	0.52 (0.48, 0.71)	0.59 (0.52, 0.86)	0.363
	**A**+**T-**			**A**+**T**+		
	**With T2DM (*****n*** = **5)**	**Without T2DM (*****n*** = **7)**	***P*** **-value**	**With T2DM (*****n*** = **13)**	**Without T2DM (*****n*** = **45)**	***P*** **-value**
Qalb	7.18 (5.69, 18.21)	6.64 (6.20, 13.64)	0.855	8.24 (5.72, 15.21)	5.29 (4.29, 6.78)	**0.001**
Aβ_1 − 42_ (pg/ml)	193.78 (100.97, 431.12)	492.85 (234.63, 542.85)	0.100	324.11 (233.38, 408.31)	411.84 (268.82, 496.93)	0.148
Aβ_1 − 40_ (pg/ml)	5,099.47 (1,701.89, 6,919.74)	8,249.93 (4,888.13, 11,550.00)	0.100	6,421.48 (5,643.56, 9,760.86)	10,768.44 (7,334.30, 12,768.67)	0.051
p-tau181 (pg/ml)	24.73 (16.31, 42.00)	43.76 (42.30, 49.95)	0.068	57.98 (54.16, 93.09)	59.60 (50.33, 91.47)	0.823
t-tau (pg/ml)	268.98 (188.87, 318.54)	367.11 (190.09, 368.57)	0.361	399.33 (230.27, 589.49)	543.69 (438.94, 745.80)	0.027
Aβ1-42/Aβ1-40	0.06 (0.04, 0.07)	0.05 (0.05, 0.07)	0.314	0.04 (0.03, 0.05)	0.04 (0.04, 0.05)	0.730
T-tau/Aβ1-42	1.39 (0.69, 2.22)	0.75 (0.67, 1.06)	0.201	1.18 (0.68, 2.97)	1.55 (1.03, 2.82)	0.244

Since there were only two patients in the “A-T-/without T2DM” group, the data was displayed with mean (SD); while data in other groups did not meet normal distribution, and was displayed with median (IQR).

CSF, cerebrospinal fluid; Qalb, cerebrospinal fluid/serum albumin quotient; AD, Alzheimer's disease; T2DM, type 2 diabetes mellitus; Aβ, β-amyloid; p-tau181, phosphorylated tau181; t-tau, total tau; SD, standard deviation; IQR, interquartile range.

### Associations among the Qalb, neuropathological biomarkers of AD and chronic vascular risk factors

The associations among the Qalb, neuropathological biomarkers of AD, and chronic vascular risk factors in dementia patients were analyzed based on correlation and linear regression models in Table 4 and Supplementary material.

**Table 4:** Associations between Qalb and neuropathological biomarkers of AD or chronic vascular risk factors.

		**Linear regressions**						
		**B**	**SE**	**Beta**	**t**	**P2-value**	**95%CI**	
							**Lower bound**	**Upper bound**
CSF AD neuropathological biomarkers	Aβ_1 − 42_	−20.775	7.737	−0.257	−2.685	0.009	−36.150	−5.399
	Aβ_1 − 40_	−305.417	105.313	−0.301	−2.900	0.005	−514.705	−96.129
	p-Tau181	−1.498	0.981	−0.165	−1.527	0.130	−3.447	0.452
	t-Tau	−12.381	7.656	−0.169	−1.617	0.109	−27.596	2.833
	Aβ_1 − 42_/Aβ_1 − 40_	−0.001	0.001	−0.131	−1.268	0.208	−0.002	0.000
	t-Tau/Aβ_1 − 42_	0.072	0.039	0.188	1.841	0.069	−0.006	0.149
APOE ε4 allele		−1.893	1.073	−0.174	−1.765	0.081	−4.024	0.239
Blood pressure	HT	−0.360	0.966	−0.037	−0.372	0.711	−2.279	1.560
	SBP	−0.011	0.027	−0.043	−0.421	0.675	−0.066	0.043
	DBP	−0.015	0.038	−0.040	−0.404	0.687	−0.091	0.060
Blood glucose	T2DM	3.382	0.932	0.339	3.630	< 0.001	1.531	5.234
	GHb	1.163	0.266	0.398	4.366	< 0.001	0.634	1.692
	FBG	1.443	0.324	0.429	4.455	< 0.001	0.799	2.087
CVD	CVD	−0.690	1.216	−0.058	−0.568	0.572	−3.106	1.725
	Fazakes	0.086	0.568	0.015	0.152	0.880	−1.043	1.215
Blood lipid	HLP	−1.049	0.944	−0.115	−1.111	0.270	−2.925	0.827
	TG	0.531	0.606	0.089	0.876	0.383	−0.674	1.736
	CHO	−0.326	0.399	−0.087	−0.815	0.417	−1.119	0.468
	HDL	−2.069	1.127	−0.195	−1.836	0.070	−4.308	0.171
	LDL-c	−0.008	0.014	−0.059	−0.591	0.556	−0.037	0.020
	BMI	−0.222	0.121	−0.183	−1.838	0.069	−0.462	0.018
Blood Hcy	HHcy	−0.751	0.942	−0.084	−0.797	0.427	−2.624	1.121
	Hcy	−0.021	0.065	−0.034	−0.322	0.748	−0.149	0.107
	FA	0.134	0.092	0.150	1.461	0.148	−0.048	0.317
	VitB12	0.001	0.001	0.069	0.680	0.498	−0.001	0.003
	Fer	−0.004	0.003	−0.133	−1.263	0.210	−0.010	0.002
Habits of SA		0.044	1.219	0.004	0.036	0.971	−2.378	2.466

The linear regression models were analyzed after adjusting gender, age, education, course of disease, and diagnosis. The variables of HT, T2DM, CVD, HLP, and HHcy meant the history of these diseases. ^*^p < 0.05, ^**^p < 0.01, ^***^p < 0.001.

CSF, cerebrospinal fluid; AD, Alzheimer's disease; Aβ, β-amyloid; p-tau181, phosphorylated tau181; t-tau, total tau; Qalb, cerebrospinal fluid/serum albumin quotient; APOE, Apolipoprotein E; HT, hypertension; SBP, systolic blood pressure; DBP, diastolic blood pressure; T2DM, type 2 diabetes mellitus; GHb, glycosylated hemoglobin; FBG, fasting blood glucose; CVD, cardiac-cerebral vascular Disease; HLP, hyperlipidemia; TG, triglyceride; CHO, cholesterol; HDL, high-density lipoprotein cholesterol; LDL-c, low-density lipoprotein cholesterol; HHcy, hyperhomocysteinemia; Hcy, homocysteine; FA, folic acid; VitB12, vitamin B12; Fer, ferritin; SA, smoking and/or alcohol consumption.

The results showed the Qalb was negatively associated with the levels of Aβ1-42 (B = −20.775, 95% CI: −36.150 – −5.399, *p* = 0.009) and Aβ1-40 (B = −305.417, 95% CI: −514.705 – −96.129, *p* = 0.005) but not the levels of *p*-tau181, t-tau, or the ratios of Aβ1-42/Aβ1-40 or t-tau/Aβ1-42. Furthermore, the Qalb was positively associated with the presence of T2DM (B = 3.382, 95% CI: 1.531–5.234, *p* < 0.001), the levels of GHb (B = 1.163, 95% CI: 0.634–1.692, *p* < 0.001) and FBG (B = 1.443, 95% CI: 0.799–2.087, *p* < 0.001) after adjusting for gender, age, educational level, course of disease, and diagnosis. While there were no significant associations among the Qalb and APOE ε4 allele, blood pressure, CVD, blood lipids, blood Hcy, or the habits of smoking and/or alcohol consumption.

**Figure 4:**
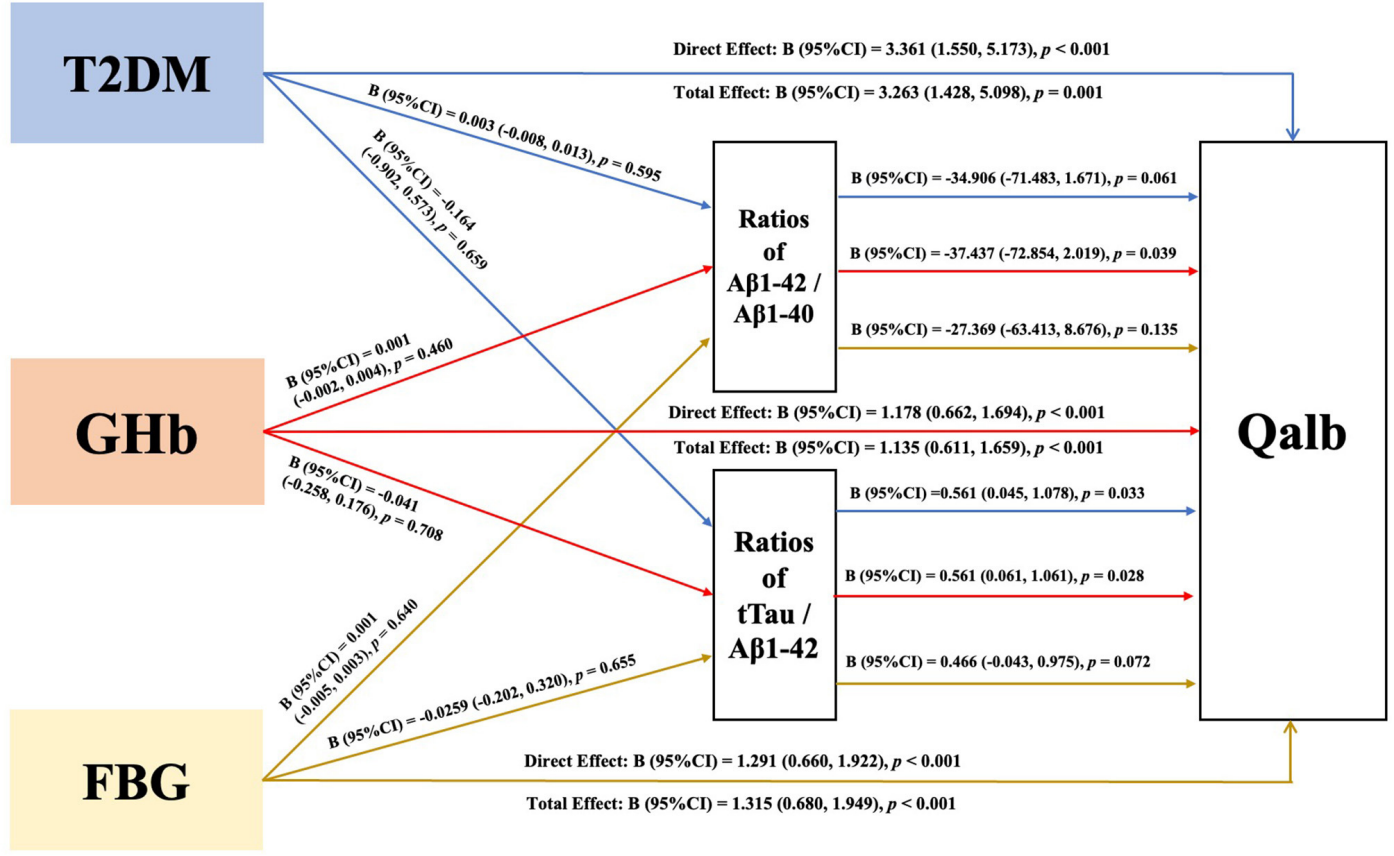
The interrelations of Qalb (Y), neuropathological biomarkers of AD (M) in CSF, and glucose exposure (X). Through the mediating effect analysis by linear regression models after adjusting gender, age, course of disease, apolipoprotein ε4 carrier, and diagnosis. All data was described as “unstandardized coefficients (B) with 95%CIs”. AD, Alzheimer's disease; T2DM, type 2 diabetes mellitus; GHb, glycosylated hemoglobin; FBG, fasting blood glucose; 95%CIs, 95% confidential intervals; Aβ, β-amyloid; t-tau, total tau; Qalb, cerebrospinal fluid/serum albumin quotient.

To determine the mediating effect of neuropathological biomarkers of AD on the association between the Qalb and glucose exposure, the specific indirect effects were investigated. GHb was a direct chronic vascular risk factor for higher Qalb (total effect B = 1.135, 95% CI: (0.611–1.659), *p* < 0.001). Ratios of Aβ1-42/Aβ1-40 or t-tau/Aβ1-42 were mediators of the association between Qalb and GHb. The direct effect of GHb on the Qalb was 1.178, 95% CI: 0.662–1.694, *p* < 0.001). All direct, total indirect, and specific indirect effects of T2DM and FBG on the Qalb are shown in Figure 4.

## Discussion

In the present study, the association between BBB permeability and the Qalb, neuropathological biomarkers of AD, and chronic vascular risk factors was investigated in a cohort of patients with different types of dementia. Results showed that chronic vascular risk factors could influence the Qalb and neuropathological biomarkers of AD. In particular, the Qalb was associated with coexisting T2DM, glucose exposure could directly or indirectly affect the integrity of the BBB through Aβ and tau in this limited number of patients.

### Qalb in dementia

Post-mortem brain tissue, neuroimaging, and CSF biomarkers from patients with AD or other neurodegenerative disorders have shown BBB disruption (Sweeney et al., 2018), and the Qalb is considered an indirect measurement of the BBB permeability. Reports are conflicting whether BBB damage can be associated with dementia and differ among subtypes. Reports of increased Qalb in AD, DLB, FTLD, and VaD compared with controls have been published (Llorens et al., 2015; Janelidze et al., 2017; Skillbäck et al., 2017; Musaeus et al., 2020), as well as reports where no difference in BBB integrity was found compared with controls (Bien-Ly et al., 2015; Olsson et al., 2016). In addition, higher BBB damage was reported in patients with VaD compared with AD or LBD patients (Skillbäck et al., 2017), however, conflicting results showed patients with DLB and VaD had a higher Qalb than AD and FTLD patients (Musaeus et al., 2020). In the present study, significant difference in the Qalb was not found among AD, LBD, and FTLD patients, however, the effect size in this study was small.

### Effects of Qalb on dementia

Previous evidence indicated that vascular pathology, including cerebrovascular disease, lacunae and multiple microinfarcts indicative of small vessel disease, hemorrhage, atherosclerosis, arteriolosclerosis, and cerebral amyloid angiopathy (CAA), was a main cause of BBB dysfunction (Llorens et al., 2015; Musaeus et al., 2020; Wong et al., 2022). Superficial siderosis (Zonneveld et al., 2014) and CMBs (Yates et al., 2014) were shown prevalent in AD, indicating the important role of vascular pathology in the pathogenesis of AD. The CMBs in AD, LBD, and FTLD patients were common, however, due to the limited sample size in the present study, a difference was not found in prevalence among the three types of dementia.

Vascular disruption can be influenced by APOE ε4 allele and chronic vascular risk factors, and might act independently and/or synergistically with Aβ to promote AD pathology (Sweeney et al., 2018). In the present study, the Qalb was slightly higher in patients with hypertension, CVD, HLP, or who smoked and/or consumed alcohol than in subjects without hypertension, CVD, HLP, or who did not smoke and/or consume alcohol. In addition, significant difference was found between patients with and without a history of T2DM. Furthermore, a higher Qalb was associated with the presence of T2DM as well as higher GHb and FBG levels after adjusting for all confounders in dementia cases. In several studies, diabetes reportedly led to the impairment of BBB integrity and subsequent BBB permeability increase in *in vivo* and *in vitro* models, which is in agreement with our results (Hawkins et al., 2007; Banks, 2019; Zhao et al., 2019). Cai *et al*. provided *in vivo* evidence that db/db mice (an animal T2DM model) have significant BBB impairments even at a young age (Cai et al., 2021). Janelidze et al. (2017) reported a higher Qalb in individuals with T2DM compared with subjects without T2DM in two different cohorts with a total of 1,015 subjects, and the authors demonstrated that T2DM was associated with high CSF levels of intercellular adhesion molecule-1 (*p* < 0.001), vascular cellular adhesion molecule-1 (*p* = 0.007), and vascular endothelial-derived growth factor (*p* = 0.024), CSF biomarkers of angiogenesis and endothelial dysfunction. Individuals with T2DM also showed increased BBB permeability in basal ganglia (Starr et al., 2003), hippocampus, occipital lobe, and frontal lobe (Abuhaiba et al., 2018) based on DCE-MRI. Furthermore, significant correlations were found between occipital (R = 0.612, *p* = 0.013) or frontal *K*trans (R = 0.579, *p* = 0.019) and GHb level (Abuhaiba et al., 2018), a marker indicating the long-term status of blood glucose. Wang et al. (2020) reported the BBB breakdown in the hippocampus, white matter, and cortex inferior temporal gyrus in syphilis individuals with high GHb levels.

CMBs did not significantly affect the Qalb and CSF neuropathological biomarkers of AD in the present study. Although clear evidence exists for the role of CMBs in the pathogenesis of BBB in AD, whether this effect is widespread in DLB and FTLD has not been investigated in many studies (De Reuck et al., 2012; Mendes et al., 2021). Llorens et al. demonstrated the Qalb in DLB negatively correlated with CSF Aβ1-42 levels but not with t-tau and p-tau levels (Llorens et al., 2015). Hijazi et al. (2022) found no association between the presence of CMBs and cortical Aβ deposition on PET imaging. Thus, the role of CMBs in different subtypes of dementia is controversial and needs further investigation.

Traditionally, APOE ε4 allele involves and accelerates BBB breakdown through the proinflammatory cyclophilin A-matrix metalloproteinase-9 (CypA-MMP9) pathway activated by brain capillary pericytes (Bell et al., 2012; Halliday et al., 2013; Montagne et al., 2020, 2021). Montagne et al. (2020) found APOE ε4 carriers (ε3/ε4 and ε4/ε4) had obvious BBB breakdown in the hippocampus and medial temporal lobe compared with non-carriers (ε3/ε3) and suggested the breakdown of the BBB contributes to APOE ε4-associated cognitive decline independently of AD pathology. Moreover, prior studies including animal models (Nishitsuji et al., 2011; Bell et al., 2012; Montagne et al., 2021; Jackson et al., 2022; Liu et al., 2022), human neuropathological studies (Salloway et al., 2002; Zipser et al., 2007; Halliday et al., 2016), molecular biomarkers of BBB damage in CSF [like sPDGFRβ (Montagne et al., 2020)], and MRI neuroimaging biomarkers (Zonneveld et al., 2014; Montagne et al., 2020; Moon W. J. et al., 2021) had clearly shown that APOE ε4 allele contributed to or enhanced BBB breakdown through synaptic plasticity compromission, or dysregulation of astrocytic end foot interactions with vessels and other ways in AD. Furthermore, permanent CMBs were shown sensitive markers indicating BBB dysfunction, and the APOE ε4 genotype can significantly increase the prevalence of CMBs (Yates et al., 2014), or promote the AD pathology toward BBB dysfunction by modulating inflammation markers in AD (Riphagen et al., 2020). However, in the present study with a relatively small sample and detected by Qalb, the association was not found between APOE ε4 allele and the Qalb. Karch et al. (2013), Janelidze et al. (2017) demonstrated similar findings and suggested APOE ε4 genotype and BBB damage were not significantly directly associated. The inconsistencies in these findings may be due to the small study cohort and because the patients were from a single institution and diagnosed with AD, LBD, or FTLD with moderate dementia severity. Furthermore, blood pressure (hypertension, systolic blood pressure, and diastolic blood pressure), CVD (Fazekas score), blood lipid profile (HLP, levels of triglyceride, cholesterol, HDL-C, LDL-C, and BMI), blood Hcy (HHcy, levels of Hcy, serum folate, serum vitamin B12, and ferritin), and the smoking habits and/or alcohol consumption were not associated with the Qalb in this study. In several studies, arterial hypertension (Santisteban et al., 2020), lipids, lipoproteins, apolipoproteins (Rhea and Banks, 2021), and HHcy (Kamath et al., 2006) were suggested to be involved in BBB disruption, which was inconsistent with the results of the present study, thus, their role in BBB function needs further investigation.

In agreement with earlier studies (Burgmans et al., 2013; Nation et al., 2019; Park et al., 2019; Riphagen et al., 2020), significant associations were observed between BBB and neuropathological biomarkers of AD in dementia subjects in the present study. The Qalb was negatively associated with Aβ1-42 and Aβ1-40, and positively correlated with the t-tau/Aβ1-42 ratio in dementia patients. Riphagen et al. (2020) reported BBB dysfunction was associated with greater AD pathology in APOE ε4 carriers. Neuropathological evidence showed that Aβ and tau pathology are not specific for AD but are present in other subtypes of dementia and in normal aging. Normally, the BBB can facilitate the clearance of Aβ and tau *via* the cerebrovascular system. In addition to the toxic effects of increased AD biomarker deposition and accumulation that induce a breakdown of the BBB, BBB disruption can disturb this pathway and enhance Aβ aggregation and tau deposition. Furthermore, the interactive and facilitative effects between neuropathological biomarkers of AD and BBB disruption cause oxidation, proinflammatory signaling, and endothelial damage to further negatively affect the pathway (Cai et al., 2018; Michalicova et al., 2020; Custodia et al., 2021; Kurz et al., 2022).

### Associations among the Qalb, AD biomarkers, and chronic vascular risk factors

The results of the present study indicated an uncertain relationship between T2DM and CSF neuropathological biomarkers of AD. Dementia patients with a history of DM had lower tau levels (both p-tau and t-tau) than subjects without a history of DM. However, significant associations between history of T2DM, FBG, or GHb levels and the ratios of Aβ1-42/Aβ1-40 or t-tau/Aβ1-42 were not found in the present study based on partial correlation analysis after adjusting for confounders such as gender, age, educational level, course of disease, and diagnosis. In addition to the association between history of hypertension and Aβ1-42/Aβ1-40 ratio, associations were not observed between Aβ1-42/Aβ1-40 or t-tau/Aβ1-42 ratios and HLP, HHcy, CVD and the smoking habits and/or alcohol consumption in dementia patients.

Based on the two-hit vascular hypothesis of AD, damage to blood vessels is the initial insult, causing BBB dysfunction and diminished brain perfusion that consequently leads to neuronal injury and Aβ accumulation in the brain (Murray-Stewart et al., 2013). Blood glucose was hypothesized to affect the neuropathological biomarkers of AD, thus affecting BBB function in dementia patients which was confirmed in the present study using human mediation models. Blood glucose (including the history of T2DM, FBG and GHb levels) was shown to affect the AD biomarkers (t-tau/Aβ1-42 ratio) and indirectly regulate the permeability of BBB. The T2DM-caused BBB dysfunction played a critical role in the pathology of neurological complications. Recent experimental results (Zhao et al., 2019) showed that histone deacetylase 3 (HDAC3) expression and activity were significantly increased in the hippocampus and cortex of db/db mice, and its activity/mRNA levels positively correlated with proinflammation, poor glycemic control, and insulin resistance. Reportedly, HDAC3 inhibition regulates Keap1/Nrf2 balance by modulating the miR-200a expression, which binds to the 3′-terminal regions of the Keap1 mRNA to downregulate its translation (Zhang et al., 2018). The reduced Keap1 level leads to an increase in Nrf2 nuclear translocation (Nrf2 dysregulation) (Montagne et al., 2015), subsequently increasing the transcription of antioxidant and anti-inflammatory genes, and mediating oxidative/inflammatory stress-induced neurovascular dysfunction and BBB disruption. Furthermore, the increased transendothelial permeability and reduced junction protein expression were found in T2DM insult *in vitro* (Zhao et al., 2019). The HDAC3 inhibition significantly attenuated the transendothelial permeability and junction protein downregulation due to HDAC3 inhibition-mediated miR-200a/Keap1/Nrf2 signaling pathway and downstream targeting junction protein expression.

### Strengths and limitations

Although some clinical evidence has indicated that BBB permeability is associated with AD biomarkers or chronic vascular factors (including history of T2DM, FBG, and GHb) involved in dementia, this is the first study in which all the variables were included in a comprehensive analysis to investigate the relationship between the three types of dementia. The results showed blood glucose, rather than other chronic vascular factors, could affect BBB permeability in patients with dementia by directly or indirectly regulating AD biomarkers.

The present study had several limitations. We only use the single parameter of BBB dysfunction measurement (Qalb), there were many other important biomarkers reflecting BBB function, such as sPDGFRβ, were not evaluated. Elevated sPDGFRβ in CSF was shown to indicate pericyte injury and BBB breakdown and predict future cognitive decline in APOE ε4 carriers but not in non-carriers independent of AD pathology (Farrall and Wardlaw, 2009; Nation et al., 2019; Montagne et al., 2020). This is similar to our findings demonstrating an indirect role of APOE ε4 in BBB. However, this was a retrospective study and all data were derived from hospitalized medical records in the cognitive impairment inpatient department, lacking CSF samples for further testing and an age-matched cognitively normal control group. The small study cohort may be the main reason why our results are inconsistent with previous relevant literature. Thus, the results require further validation in a larger study population with multiple diagnoses. In addition, the Qalb, an easy to assess indicator commonly used in practice but still affected by age and other factors, was used to reflect BBB permeability. Although adjustments were made for the potentially confounding effects of age when analyzing the data, the contribution of CSF turnover to the Qalb cannot be entirely excluded. However, in several reports regarding different research topics (Chen, 2011; Castellazzi et al., 2020), the albumin ratio was still considered a robust and reliable standard surrogate marker used to measure BBB integrity in epidemiological studies and daily practice, and has been shown to accurately reflect BBB integrity. Altogether, direct assessments of BBB permeability and function, such as using DCE- MRI and specifically labeled tracers, are warranted to confirm the results of the present study.

## Conclusion

In the present study, clinical evidence showed that chronic vascular risk factors could influence the BBB function and neuropathological biomarkers of AD in dementia patients. Glucose exposure could directly or indirectly affect the integrity of the BBB through Aβ and tau, however, the APOE ε4 allele, CVD, HLP, HHcy, or smoking habits and/or alcohol consumption did not show significant effect on the Qalb which might be due to the study patients and small sample-size cohort. The results indicate glucose stability plays an important role in dementia protection and management. Future studies with large number of dementia cases are necessary, and should be performed in which the molecular mechanisms underlying the effect of glucose on BBB breakdown are investigated and therapeutic interventions explored.

## Data availability statement

The raw data supporting the conclusions of this article will be made available by the authors, without undue reservation.

## Ethics statement

The studies involving human participants were reviewed and approved by this study was performed according to the Helsinki Declaration and approved by the Ethical Review Board of Beijing Tiantan Hospital (KYSQ 2021-068-01). The patients/participants provided their written informed consent to participate in this study.

## Author contributions

YJ had full access to all of the data in the study and take responsibility for the integrity of the data and the accuracy of the data analysis. Concept and design were performed by YJ and XL. JG wrote the first draft of the manuscript. XY, GZ, and XL contributed to the critical revision of the manuscript for important intellectual content. Statistical analysis was performed by JG and SL. Fundings were obtained from YJ. All authors contributed to collect medical records, the acquisition, analysis, or interpretation of data. All authors accept responsibility for all aspects of the manuscript, read, and approved the final version of the manuscript.
